# Nutritional status, diet and viral respiratory infections: perspectives for severe acute respiratory syndrome coronavirus 2

**DOI:** 10.1017/S0007114520003311

**Published:** 2020-08-26

**Authors:** Ana Heloneida de Araújo Morais, Jailane de Souza Aquino, Juliana Kelly da Silva-Maia, Sancha Helena de Lima Vale, Bruna Leal Lima Maciel, Thaís Sousa Passos

**Affiliations:** 1 Nutrition Postgraduate Program, Center for Health Sciences, Federal University of Rio Grande do Norte, Natal, RN 59078-970, Brazil; 2 Department of Nutrition, Center for Health Sciences, Federal University of Rio Grande do Norte, Natal, RN 59078-970, Brazil; 3 Nutrition Postgraduate Program, Center for Health Sciences, Federal University of Paraíba, João Pessoa, PB 58050-085, Brazil

**Keywords:** Malnutrition, Obesity, Ultra-processed foods, COVID-19, Inflammation, ACE2, angiotensin-converting enzyme 2, COPD, chronic obstructive pulmonary disease, DII, dietary inflammatory index, SARS-CoV-2, severe acute respiratory syndrome coronavirus 2, TLR, Toll-like receptor

## Abstract

COVID-19, caused by severe acute respiratory syndrome coronavirus 2 (SARS-CoV-2), was recognised by the WHO as a pandemic in 2020. Host preparation to combat the virus is an important strategy to avoid COVID-19 severity. Thus, the relationship between eating habits, nutritional status and their effects on the immune response and further implications in viral respiratory infections is an important topic discussed in this review. Malnutrition causes the most diverse alterations in the immune system, suppressing of the immune response and increasing the susceptibility to infections such as SARS-CoV-2. On the other hand, obesity induces low-grade chronic inflammation caused by excess adiposity, which increases angiotensin-converting enzyme 2. It decreases the immune response favouring SARS-CoV-2 virulence and promoting respiratory distress syndrome. The present review highlights the importance of food choices considering their inflammatory effects, consequently increasing the viral susceptibility observed in malnutrition and obesity. Healthy eating habits, micronutrients, bioactive compounds and probiotics are strategies for COVID-19 prevention. Therefore, a diversified and balanced diet can contribute to the improvement of the immune response to viral infections such as COVID-19.

COVID-19, caused by severe acute respiratory syndrome coronavirus 2 (SARS-CoV-2), was recognised by the WHO as a pandemic in 2020^(1)^. The physiological and immunological processes that trigger COVID-19 clinical features are still not wholly unveiled for sufficient therapy identification and planning of effective therapies^(2)^.

In the initial pandemic period, research mostly explored epidemiology, causes, clinical features, diagnosis, prevention and control strategies regarding SARS-CoV-2. Despite the high relevance of these studies, more investigations are necessary to validate protocols to manage this public health emergency in the short and long term^(3)^. There has been more scientific data available alongside the coronavirus dissemination, and the knowledge has consolidated in several areas. However, studies regarding prevention, besides those involving the importance of social isolation measures, sanitisation of superficies and hands and the personal protection equipment use, are scarce^(4)^.

The host’s preparation to combat the virus is an important strategy to avoid COVID-19 worsening, especially considering the inflammatory status and, consequently, the immune system reinforcement^(5–7)^.

Inflammation plays a crucial role in injuries caused by SARS-CoV-2. The release of inflammatory cytokines, in cases of COVID-19, leads to a cytokine storm, generating an immune dysregulation initiated by T cells and inflammatory monocytes^(8)^. Thus, the severity of COVID-19 has been associated with an underrecognised hyperinflammatory status characterised by increased TNF-*α*, IL-2, IL-7, macrophage inflammatory protein 1-*α* (CCL3), granulocyte colony-stimulating factor and interferon-*γ* inducible protein 10 also known as C-X-C motif chemokine 10^(9)^.

Therefore, the virus damage is intensified by an exacerbated immune response, which can lead to destructive consequences to several human organs, such as the lungs, kidneys, heart, liver and other organs from the gastrointestinal tract^(2,8,9)^. Therefore, the coronavirus triggers an inflammatory response, and the host’s immune system can itself be responsible for worsening of the COVID-19 outcomes. Thus, controlling the inflammatory response seems to be an essential strategy in combating the virus. According to Mehta *et al.*^(10)^, all patients with severe COVID-19 should be screened for hyperinflammation for whom immunosuppression could improve mortality.

On the other hand, the main functions of the innate and adaptive immune systems are to recognise aggressor agents and to activate effector mechanisms, defending the organism against infections, besides combating many inflammatory diseases^(11)^. Concurrent with the pro-inflammatory response, anti-inflammatory mechanisms are stimulated to avoid very aggressive inflammation. Thus, the immune system gives a persistent tolerance status, and without it, more severe damage and organ failure could commonly occur^(12)^.

This immune system defence ability can be programmed through epigenetic and metabolic programming of the innate response, which modulates the effector immunity. These changes can lead to responses with qualitative and quantitative adjustments, consequently reducing the risk of secondary infections and other diseases related to a reduction in the immune system activity^(12)^.

Modern life, with daily stress, sleep deprivation and an unbalanced diet, can contribute to the initiation of a chronic low-inflammation status, which can negatively affect the immune system^(13,14)^. Concomitantly, the global population has been under stressful situations due to the SARS-Cov-2 ongoing pandemic. According to Slavich^(15)^, social ties’ maintenance is a fundamental organiser principle of human behaviour, and threats to social security are critical characteristics of psychological stressors that raise the risk of diseases; hence, lifestyle interventions that mitigate the inflammation and support the immune system, such as a healthy diet adhesion, have a primordial role in combatting infections and disease installation^(16)^. Nutritional status is also directly related to inflammation and, consequently, with the immune response. Moreover, malnutrition and obesity are severe public health issues and need attention^(17–19)^.

Accordingly, diets with anti-inflammatory profiles are associated with healthy and nutritionally balanced food choices, aiming at weight maintenance, cell stress reduction and the organism’s proper functionally^(14,20)^. These diets have been shown to support the immune system to prevent and combat opportunistic infections, consequently, modulating the severity of these diseases during the COVID-19 pandemic crisis^(5,6)^. Therefore, healthier food choices can contribute to this modulation.

Several diets can reduce inflammation through the stimulus of anti-inflammatory food intake and control or exclusion of pro-inflammatory foods^(16,20,21)^. There are specific features associated with the individual response, and this should be considered to distinguish these nutritional orientations and recommendations.

As reviewed by Christ *et al.*^(21)^, inflammation is linked to the intake of a Western diet, and some signs of chronic inflammation or meta-inflammation include the excess of visceral fat (abdominal fat), digestive problems, headache, eczema, high blood pressure, hypercholesterolaemia, and joint pain^(21)^. Additionally, this inflammation can contribute to the development of many noncommunicable diseases, such as cardiac diseases, autoimmune conditions and diabetes^(21)^.

The existing link among nutritional status, inflammation, viral respiratory infections and food choices becomes essential. Thus, this review presents perspectives on preventing and improving the immunologic response during viral infections, with perspectives for COVID-19.

## Malnutrition, obesity and the increased risk for viral respiratory infections

Malnourishment, specifically undernutrition and overweight/obesity, alters the immune response, increasing the risk of infections from several pathogens^(22)^, such as influenza and coronavirus^(23–28)^. The perception that nutritional status is associated with the response to infections is relatively recent. The first review of the WHO on the topic was published in 1968 from works developed by Nevin Scrimshaw’s group. Two forms of interaction between malnutrition and infections were proposed: synergistic and antagonistic^(29)^.

The synergistic interaction proposed that an infection could aggravate malnutrition and that malnutrition could decrease resistance to infection, with the simultaneous presence being worse for the host. Thus, the notion that the infection could worsen malnutrition and vice versa in a vicious cycle was established. On the other hand, the antagonistic interaction proposed that, in some exceptional cases, malnutrition could decrease the multiplication of the infectious agent. Thus, the combined effect of infection and malnutrition would be less than expected^(29,30)^.

Several studies have shown that malnutrition causes changes in innate and adaptive immune responses, leading to greater susceptibility to infections^(31–33)^. One of the most evident changes is the involution of the thymus, structurally and functionally, reducing the T cell response^(33–36)^. All complement components (except C4) are reduced in malnourished patients, especially C3 and factor B^(37,38)^. Studies have also shown, as discussed by Cunningham-Rundles *et al.*^(39)^, that malnutrition affects phagocytic function and the production of cytokines and antibodies.

Despite the strong evidence of associations between malnutrition and infections, the mechanisms driving this vulnerability are not fully understood^(40–42)^. This lack of consensus is possibly explained by the complex interactions between nutritional status and infection that result in a vicious cycle^(29)^.

In this vicious cycle, infections induce an inflammatory response (production of IL-1, IL-6, TNF-*α*, PGE2) that commonly generates fever, increased catabolism, appetite loss and alteration of intestinal absorption. These alterations increase nutritional demands and, added to appetite loss, induce or aggravate malnutrition^(41,43)^. Malnutrition, in turn, increases the risk of infection by reducing gut barrier function^(41,44)^, modifying the intestinal microbiota^(45)^, compromising immune cell generation and activation, altering the regulation of inflammatory adipocytokines and limiting the uptake of macro- and key micronutrients^(46,47)^.

Undernutrition also decreases the lipid tissue, altering adipokine production, ultimately leading to innate and adaptive immunity restrictions. In malnutrition, leptin production decreases, while adiponectin increases. Adiponectin promotes the activity of alternative macrophages (M2), with the secretion of anti-inflammatory cytokines IL-10 and IL-1R*α*, reducing T cell responses and B cell production. Reduced leptin and increased adiponectin in malnutrition combined with alterations in stress hormones production, such as cortisol, limit the ability of immune cells of proper pro-inflammatory responses^(47)^.

The up-regulation of glycolytic pathways with restriction of oxidative phosphorylation for the high metabolic needs of pro-inflammatory effector cells is compromised in malnutrition. These metabolic constraints limit T and B cell activation and proliferation and preferentially activate regulatory T cells that rely on alternative energy sources, such as fatty acid oxidation^(47,48)^.

Macrophages and neutrophils’ proliferation and capacity to infiltrate in the infection sites, phagocytosis and kill bacteria through the production of reactive oxygen species is also limited in these metabolic restrictions. Ultimately, the pro-inflammatory cytokines, essential to pathogen containment and killing, TNF-*α*, IL-6 and IL-8 are reduced in malnutrition, while the anti-inflammatory cytokines IL-10 and IL-33 are increased^(47)^.

Thus, undernutrition causes the most diverse alterations in the immune system, leading to increased susceptibility to infections. Although there is a vast literature on diarrhoeal, bacterial pneumonia and measles concerning their associations with malnutrition^(40–42)^, for viral lower respiratory tract infections, data are still limited, and mostly related to children.

In all-cause lower respiratory tract and respiratory syncytial virus infections, undernourished children presented a higher risk for severe diseases^(49,50)^. Most of the studies were experimental for influenza infections and have shown that protein and energy malnutrition increased the risk for severe infections^(23,51)^, reducing virus-specific antibody and CD8+ T cell responses^(23)^. Experimental studies suggest that malnutrition might facilitate the emergence of viral variants with more pathogenic potential than the original strain^(24)^.

Since the H1N1 influenza pandemic in 2009, there has been an increase in the number of investigations regarding the relationship between worsening respiratory infections and body mass index^(52,53)^. It has been found that obesity is an independent risk factor for the severity of the disease, the length of hospitalisation and increased mortality^(53,54)^.

Complications resulting from viral infections with the influenza virus in obese people have also been described in coronavirus infections, such as SARS and MERS^(25)^. Obesity results in a decreased immune response to infectious agents, and severe obesity (BMI of 40 kg/m^2^ or above) increases the risk of acute respiratory distress syndrome, leading to worse post-infection results^(53,55)^.

Obesity *per se* can complicate lung mechanics by restricting lung volume. The pulmonary tissue is the most sensitive human tissue in critical COVID-19 patients. The progression of respiratory viral infections in obese patients includes extensive viral replication in the lung, progression to viral pneumonia and prolonged and increased viral shedding^(53)^. Among the mechanisms that contribute to the increased susceptibility of these patients, there are non-immune factors and also mechanisms from the immune system activity attenuation to chronic inflammation^(25,52)^.

Besides, metabolic dysregulation caused by excess adiposity compromises the immune system and the host’s defence mechanisms^(54)^. There is a direct link between chronic inflammation and the cytokine storm that favours the respiratory distress syndrome of patients with COVID-19^(56)^. The virus’s entry into the cell is mediated by ectoenzyme angiotensin-converting enzyme 2 (ACE2) located on the lungs’ cell surface and by the serine protease TMPRSS2. When there is inhibition of this enzyme, pro-inflammatory responses and stimulation of aldosterone secretion occur, with a consequent increase in vascular permeability^(57)^.

Mahmudpour *et al.*^(58)^ highlighted that this negative regulation of ACE2 triggers an exacerbated release of inflammatory cytokines, due to the dysregulation of the angiotensin II/angiotensin type 1 receptor (AT1R) axis. Besides, the researchers point out that the infection caused by the new coronavirus also causes changes in three other axes, which may be directly involved in the cytokine storm, responsible for the worsening of the clinical condition of infected patients. These axes are related to the attenuation of the Mas (ACE2/MasR axis), the highest activation of the ACE2/bradykinin BK1R/des-Arg9-bradykinin axis and activation of the complement system, which is related to complement factor 5a (C5a) and C5b-9.

Pinheiro *et al.*^(18)^ observed that the expression of angiotensinogen, angiotensin-converting enzyme (ACE2), IL-6 and TNF-*α* were significantly elevated in both malnutrition and obesity compared with the eutrophic group. The results suggested that the increase in inflammation in adipose tissue that generally occurs in the nutritional status of obesity also occurs in malnutrition and is associated with the regulation of the renin–angiotensin system, influencing the development of metabolic disorders. Therefore, this suggests that obese and malnourished patients, consequently, are more susceptible to develop the most severe forms of COVID-19. This evidence becomes worrying due to a recent study published by Hoffmann *et al.*^(59)^, which demonstrated the relationship between the renin–angiotensin system and the severity of SARS-CoV-2 infection because of the viral protein spike (S) uses ACE2 receptors to enter human cells and trigger infection.

The elderly have malnutrition, as observed by Li *et al.*^(60)^ in a cross-sectional study with 162 patients admitted to Wuhan Tongji Hospital from January to February 2020, diagnosed with COVID-19. They noted that 27·5 % of patients over 65 years were at risk of malnutrition, and 52·7 % were malnourished. In these cases, it was pointed out that the fear of contracting the disease, the concern with long-term social isolation and the desire to return to a routine led to anxiety, compromising appetite and aggravating malnutrition. On the other hand, obesity sharply increases the risk of hypertension, type 2 diabetes and CVD, three of the most critical conditions underlying COVID-19^(61)^.

Thus, the elderly represent a risk for aggravation and death in cases of COVID-19. This evidence is due to the part of these individuals present obesity and associated diseases, such as insulin resistance, type 2 diabetes mellitus, hypertension, CVD, kidney and liver disease. Besides, the increased risk for respiratory infections and lung diseases is directly related to the severity of COVID-19^(54)^.

Insulin resistance, diabetes and hypertension may cause imbalances in pathways regulating angiotensin-converting enzymes 1 and 2 (ACE1 and ACE2). In these conditions, the activation of angiotensin receptors (AT1R and AT2R) is increased, promoting higher pro-inflammatory responses and aldosterone secretion. Local vascular permeability increases in response, worsening the respiratory syndrome caused by SARS-CoV-2. Type 2 diabetes also induces the expression of angiotensin-converting enzymes in the liver and heart, which may contribute to higher mortality due to multiple organ failure in cases of SARS-CoV-2^(57)^.

ACE2 has a fundamental role in the cardiovascular system and is directly involved with cardiac function and hypertension development. Thus, COVID-19 patients with pre-existing CVD present worsen the risk for a severe disease that might be explained by increased ACE2 expression^(62,63)^. Acute coronary syndrome leads to cardiac functional reserve impairment. Patients with this condition, when with COVID-19, are at higher risk for myocardial infarction type 1, myocardial infarction type 2 or increased metabolic demands leading to heart failure and death^(62)^.

However, regardless of the effect of co-morbidities, excess adiposity increases the incidence of complications from mortality from respiratory causes^(54)^. Obesity is a risk factor for asthma. Although the reasons for this increased risk are not fully understood, it is believed that pro-inflammatory adipokines in obesity may augment airway inflammation, producing asthma^(64)^. Reports are still not consensual of asthma as a risk factor for severe COVID-19, which may be possibly explained to differences in asthma endotypes. SARS-CoV-2 infection induces an attenuated interferon-I and INF-III signature, similar to that observed in patients with asthma. Thus, COVID-19 is expected to trigger asthma exacerbations^(65)^.

COVID-19 produces symptoms common to other viral infections (such as fever, dry cough and dyspnoea). However, it can evolve with systemic hypoxia due to reduced pulmonary functions, increased adipokines and cytokines^(25)^. Associated with this, the white adipose tissue of obese patients produces immunomodulatory adipokines that include leptin, adiponectin and pro-inflammatory cytokines: TNF-*α*, IL-6 and IL-1*β*^(52,53)^.

Leptin is an essential regulator for T cell maturation. Leptin resistance observed in obese individuals could explain the higher susceptibility to respiratory infections, and in general, impaired immunity, as this would limit the availability of leptin function^(66)^. Similarly, individuals with a genetic mutation that prevents the adequate synthesis of leptin become morbidly obese and have weakened immune defences^(52)^.

IL-1*β* upregulates a wide range of pro-inflammatory activities in immune cells. It induces the rapid recruitment of neutrophils to infection sites, activation of endothelial adhesion molecules and induction of chemokines. It also induces the release of other cytokines (such as interferon-*γ* and TNF-*α*, for example), the proliferation of auxiliary B and T cells and improves the presentation of the antigen^(67)^.

Animal studies suggest that obesity increases the severity and duration of viral infections, increasing the potential for the evolution of pathogenic viral variants^(61)^. One of the strategies to initiate the innate immune system’s response is the recognition of molecular pathogen-associated molecular patterns through Toll-like receptors (TLR). TLR belong to the IL-1 receptor superfamily (IL-1R). TLR4 is expressed in different cell types, including adipocytes. In obese individuals, the increase in plasma fibrinogen levels can activate the TLR4 pathway inducing TNF-*α* and IL-1*β*, for example, amplifying the inflammatory response^(68)^. Thus, for COVID-19 treatment, therapeutic options include steroids, intravenous immunoglobulins, selective cytokine blockade and Janus kinase inhibition^(10)^.

According to the review by Briguglio *et al.*^(69)^, malnutrition and obesity trigger other diseases that can be virulence factors increasing the severity of COVID-19. In energy–protein malnutrition, the impaired immune cell activation leads to more remarkable viral persistence and increased trafficking of inflammatory cells to the lungs. In obesity, increased body fat generates low-grade systemic inflammation related to a leptin-induced increase in CD4 T cells. Besides, nutritional status has been implicated in altering viral evolution, as previously discussed^(24)^. Among the strategies to reduce the risk of COVID-19, both at the community and individual levels, are (1) mitigation of transmission from person to person and (2) the adoption of lifestyle practices that strengthen immunological health, such as the combination of physical activity and diet^(54,61)^.

## Eating habits and the immune response: perspectives for COVID-19

Nutritional status is directly related to inflammation and, consequently, to the immune response. As already mentioned, malnutrition and obesity are characterised as severe global public health problems and deserve attention^(17–19)^, mainly during the COVID-19 pandemic. Besides, in both undernutrition and obesity, the ingestion of monotonous diets rich in ultra-processed foods may lead to vitamin and mineral deficiencies, impairing the immune system and increasing susceptibility to SARS-CoV-2^(69)^.

During the SARS-CoV-2 pandemic, the world population is experiencing stress characterised by nervousness and, mainly, fear about the future consequences related to the effects of the disease. Social isolation is an effective strategy to reduce infections, but reduced access to family, friends and social support can increase problems such as anxiety and depression^(70)^. In this scenario, daily stress, sleep deprivation and a nutritionally unbalanced diet create a state of chronic inflammation, negatively affecting the immune system^(13,14)^. Classically, relevant inflammatory stimuli are endocrine, toxic, mechanic, genetic and metabolic factors, as well as viral and bacterial infections. However, dietary determinants and lifestyle can also induce inflammation and, consequently, impair adequate immune system functions.

Thus, diet is part of the modifiable contributors to the development and progression of chronic diseases. In this process, pro- or anti-inflammatory mechanisms are involved. Respiratory infections, for example, are characterised by airway and systemic inflammation^(71)^. In the context of COVID-19, a disease with intense inflammation, the cytokine storm generates an immune dysregulation that can lead to multiple organ failure and death^(54,56)^. Therefore, it is important to understand the role of diet in inflammation, deficiencies or excesses, and its pro- or anti-inflammatory impact.

The dietary inflammatory index (DII) has been used in investigations focused on studying the relationships of diet and inflammation. The DII is standardised through the average of world dietary intake and validated against inflammatory biomarkers^(72)^. According to Wirth *et al.*^(72)^, regarding human nutrition and health, the DII has been a useful instrument to help individuals select anti-inflammatory food and meals. This selection brings the additional benefit of reducing chronic inflammation and, consequently, leads to a lower risk of inflammation-related diseases.

A pro-inflammatory diet influences innate and adaptive immune responses and can promote allergic airway inflammation. The DII was higher in US children and adults with current asthma or wheeze. Inflammatory mediators related to obesity have been directly associated with asthma in obese people by enhancing airway inflammation^(64)^. Additionally, multivariable regression models were applied to data from 8175 children (6–17 years) and 22 294 adults (18–79 years) who participated in the 2007–2012 National Health and Nutrition Examination Survey. High fractional exhaled nitric oxide (a marker of oeosinophilic airway inflammation) was found among children with increased DII. Adults showed compromised lung function by decreased forced expiratory volume in 1 s and forced vital capacity associated with DII^(73)^. Similar findings were found between Hispanic adults for DII^(74)^.

Several foods and dietary patterns have been identified as pro-inflammatory, inducing secretion of inflammatory mediators, and free radicals. Oxidative stress can maximise deleterious effects from the inflammatory process. The most studied foods over their pro-inflammatory characteristics have been refined sugars, hydrogenated fats, poorly digested allergenic proteins, processed and ultra-processed foods, which can be rich in chemical additives and pesticides^(21,75–77)^. These foods are often present in Western diets, which have been characterised as pro-inflammatory and closely related to the functioning of the immune system^(21)^. Therefore, interventions must be made in the lifestyle of the population to reduce inflammation and strengthen the immune system. Adherence to healthy eating habits plays a crucial role in reducing inflammation and helping combat infections^(16)^, such as those triggered by SARS-CoV-2 in malnutrition and obesity.

The organism’s homoeostasis is frequently challenged by physical and psychological stresses, such as exposure to viruses, leading to an immune response. Mostly, the disruption is quickly resolved, and the equilibrium is restored. However, sometimes the damage is prolonged and intense. Nutrients are important to the proper response to body aggressions. They act from energy metabolism to inflammatory and immune functions, including the redox system. Therefore, impairment in the inflammatory response can be linked to nutritional inadequacies and cause detrimental effects on health^(78,79)^. In addition, the physiological and pathological situation can lead to suboptimal nutrition by increasing nutrient demands^(79)^.

Several diets with anti-inflammatory characteristics have a prominent role when associated with healthy, nutritionally balanced food choices aimed at maintaining weight, reducing cell stress and the full functionality of the body^(14,16,20,21)^. These diets support the immune system, preventing, protecting and combating opportunistic infections and, consequently, may modulate the severity of these diseases in times of COVID-19^(5,6)^.

Anti-inflammatory outcomes have been attributed to healthier diets, such as the Mediterranean diet. Dietary Approaches to Stop Hypertension and Harvard Healthy Eating Plate have in common the orientation of diets rich in fruits, vegetables and fish that promote lower inflammation status^(80–83)^. Other work investigated adherence to the Dietary Approaches to Stop Hypertension diet in patients with chronic obstructive pulmonary disease (COPD) in comparison with subjects without COPD^(84)^. Patients with COPD ingested lower vitamin C, vitamin E and dietary fibre. Besides, a COPD symptom, cough, was significantly decreased by high adherence to the Dietary Approaches to Stop Hypertension diet^(84)^.

Balanced nutrition is essential to prevent and manage viral infections, favouring the immune response^(85)^. Although data regarding nutritional management of SARS-CoV-2 infection are currently limited, healthier food choices support the immune system and protect against worse outcomes.

## The role of micronutrients in respiratory infections and COVID-19

Considering the diverse eating habits of the populations and their respective usual diets, intake maintenance and monitoring of micronutrients involved in immunocompetence against infections are essential, including vitamins A, C, D and E, in addition to Fe, Zn and Se. These micronutrients may be deficient in both malnutrition and obesity^(16,69)^.

Obesity is considered a state of chronic oxidative stress and inflammation. The inflammatory character of obesity is related to the increase in adipose tissue concomitant with the reduction of the expression of anti-inflammatory substances and the oxidative stress that plays an essential role in the development of co-morbidities^(86)^.

In this context, nutrition affects not only obesity itself but also the low-grade chronic inflammation and oxidative stress associated with this disease. Potentially protective dietary components, such as antioxidants, are associated with maintaining body weight, reducing the incidence of metabolic diseases, reducing low-grade inflammation biomarkers, such as serum C-reactive protein and adipokines and attenuating systemic oxidative stress and specific organs in obesity^(87)^. As we describe in the following topics, the consumption of specific micronutrients in the diet can have additional advantages against viral infections.

### Vitamin A

Vitamin A, especially *β*-carotene with pro-vitamin A activity, is very abundant in sweet potatoes, carrots and green leafy vegetables^(6)^. There is little information about dietary intake and supplementation of this vitamin to prevent viral infections such as COVID-19 in the adult population^(16)^. Despite this, Briguglio *et al.*^(69)^ reported that in malnutrition and sarcopenic obesity, deficiency of vitamin A and other vitamins is prevalent, which can increase the susceptibility to SARS-CoV-2 infection. Thus, it is essential to keep consumption within the daily recommendations^(88)^ and evaluate the need for supplementation in malnutrition and obesity as a prevention strategy.

### Vitamin C

Smoking is strongly associated with respiratory morbidities and diseases, as oxidative stress can lead to tissue damage and lung dysfunction. Omenaas *et al.*^(89)^ observed significant differences in the intake of vitamin C by current smokers. There was a lower OR for cough and wheeze in current smokers with higher dietary vitamin C intake (≥395 mg/week). The results were attributed to the antioxidant action of vitamin C on the oxidative stress caused by cigarette smoking. The oxidative imbalance in alveolar structure can lead to chronic inflammation by stimulating neutrophils and macrophages, enhancing proteases and oxidants and inactivating protease inhibitors. According to the review by Carr & Lykkesfeldt^(90)^, evidence suggests that a daily intake of at least 200 mg of vitamin C may promote beneficial health effects for smokers.

The maintenance of adequate vitamin C levels in the body is essential in preventing of COVID-19, given the antioxidant and anti-inflammatory action that protects the body from pulmonary infections, being an important immune modulator. It is also necessary for treating COVID-19 since its levels are reduced in affected people^(91)^. Despite the recommendation to consume food sources of this vitamin in the diet such as red peppers, oranges, strawberries, broccoli, mango, lemon and other fruits and vegetables^(6)^, early intravenous or oral administration of vitamin C at high doses has been recommended in the treatment of COVID-19 and used as a preventive measure for susceptible populations, without any adverse effects^(92)^.

### Vitamin D

Recommendations to limit social contact can limit exposure to the sun and physical activity, directly affecting the vitamin D status in the body^(93)^. Vitamin D insufficiency (a serum 25-hydroxy vitamin D level of <30 ng/ml) was associated with current asthma and current wheeze in children, as well as with current wheeze in adults^(94)^.

Vitamin D is known for its anti-inflammatory effects, some immune cells have vitamin D metabolite receptors and their function is affected after ligand binding. Administration reduces the expression of pro-inflammatory cytokines and increases the expression of anti-inflammatory cytokines^(95)^. Besides, vitamin D treatment inhibited lipopolysaccharide-induced p-p38 in monocytes, whereas it induced mitogen-activated protein kinase phosphatase-1 mRNA expression and glucocorticoid receptor binding and histone H4 acetylation in the glucocorticoid response element^(96)^. However, the mechanisms of vitamin D metabolite effects on respiratory virus-induced expression of cell surface markers mediating viral entry to respiratory epithelial cells are not clear^(97)^.

Supplementation (vitamin D_2_ or D_3_) seems to be plausible as a strategy to prevent moderate and severe symptoms of respiratory infections, such as COVID-19, considering the protective effects against acute respiratory infections and lung injury^(98,99)^, especially in malnutrition and obesity, in which this vitamin is deficient^(69)^.

### Vitamin E

Vitamin E can also be deficient in malnutrition and obesity and be a virulence factor for SARS-CoV-2^(69)^. Vitamin E has antioxidant and anti-inflammatory activities and a moderate effect on respiratory tract infections^(100)^. These are beneficial in malnutrition and obesity, reinforcing the need for diet consumption as prevention to COVID-19.

Vitamin E also influences respiratory tract infections^(19)^. A long-term prospective cohort study found that men with higher serum *α*-tocopherol had decreased mortality from respiratory disease and other significant mortality causes^(101)^. The daily recommendations for vitamin E are 15 mg/d for the adult population^(102)^, with vegetable oils (soya, maize, sunflower, among others), seeds, nuts, spinach and broccoli as the primary dietary sources.

Some minerals can also be involved in the prevention and adjuvant treatment of respiratory infections, such as COVID-19, considering the interaction between vitamins A, D, E and C and these minerals.

### Iron

Among the minerals in the diet, Fe is noteworthy, given the high prevalence of deficiency worldwide affects people in different life cycles and social classes. The relationship between Fe and infections is not fully understood. Previous studies have shown that Fe deficiency predisposes individuals to infections^(103)^, while others suggest protection^(104,105)^. Thus, the most prudent in preventing COVID-19 seems to be the maintenance of this mineral’s homoeostasis in the body. Regular dietary consumption of sources is essential, such as meats, viscera, eggs, beans and dark green vegetables, respecting positive interactions, especially non-haem Fe of plant origin, with other nutrients that can facilitate bioavailability (vitamins A and C).

### Zinc

Zn is another dietary component critical for sustaining proper immune function and is involved in the development and maintenance of immune cells. Zn modulates the pro-inflammatory response by targeting NF-κB. Its deficiency can disturb the inflammatory response, mainly elevating inflammation and damage to host tissue^(106)^, as in respiratory diseases. Lin *et al.*^(107)^ showed an increased prevalence of obstructive lung disorder among individuals with low Zn intake regardless of smoking status.

A recent paper suggests Zn as an adjuvant in COVID-19 management due to its potential to reduce inflammation and modulate antiviral immunity^(108)^. Some molecules evaluated for the treatment of SARS-Cov-2 infection inhibit the affinity of the viral enzyme RNA-dependent RNA polymerase. These molecules act as Zn ionophores, block mRNA capping, inhibit RNA polymerase elongation and induce mutations in viral replication^(109–111)^, consequently working against SARS-CoV-2. Thus, intracellular Zn^2+^ concentrations might be important to block SARS-CoV-2 replication.

Thus, this appears to be a healthy dietary pattern to be followed during social isolation as prevention of COVID-19. Zn is present in several foods of the Mediterranean diet, such as poultry, red meat, nuts, pumpkin seeds, sesame, beans and lentils.

### Selenium

Another micronutrient worth mentioning in the context of COVID-19 is Se, as preliminary results show a positive association between the reported cure rates for COVID-19 and the Se status in the Chinese population^(7)^, which is consistent with the evidence of the antiviral effects of Se from previous studies^(112,113)^. However, it is still too early to state what dose of Se should be consumed or supplemented for COVID-19 prevention. Virulence tests still need to be performed. In the abovementioned study, the authors did not have data such as age, co-morbidities, nutritional status and nutritional therapy of the affected patients^(7)^. Despite this, McCarty & DiNicolantonio^(114)^ suggest 50–100 µg/d as a provisional daily dosage to help control influenza and coronavirus viruses, including the daily Se recommendation, 50 µg/d^(102)^. Among the primary dietary sources of Se are fish, beef, poultry and nuts^(115)^.

## Importance of *n*-3 PUFA, phenolic compounds, fibres and probiotics in the COVID-19 scenario

The consumption of foods or supplement sources of PUFA *n*-3, phenolic compounds, fibres and probiotics deserves special attention in the COVID-19 prevention strategy^(85,114,116)^.

### *n*-3 PUFA

Based on the review study by Rogero & Calder^(68)^, scientific evidence confirms that SFA lead to an increase in the inflammatory response through the activation of the TLR4 signalling pathway. On the other hand, MUFA and PUFA do not promote the activation of this pathway. They can reduce the pro-inflammatory effect induced by a diet rich in SFA^(68)^.

In this context, the *n*-3 fatty acids EPA and DHA are critical components in inflammatory responses. They are enzymatically converted to specialised pro-resolving mediators known as resolvins, protectins and maresins, collaborating to resolve inflammation, including respiratory tract^(19,117,118)^.

According to the review by Messina *et al.*^(116)^, *n*-3 PUFA play an important role in reducing reactive oxygen species and pro-inflammatory cytokines, such as TNF-*α*, IL-1*β*, IL-6 and IL-8. They can reduce the activation of NF-κB to stimuli such as stress, cytokines, free radicals, ultraviolet radiation, oxidation of LDL and antigens. Thus, they can be used to treat metabolic, cardiac, inflammatory and autoimmune diseases.

A recent study assessed *n*-3 fatty acid intake and morbidity in respiratory diseases marked by inflammation in the US population, specifically adults (*n* 878) with COPD, a chronic inflammatory lung disease causes obstructed airflow in the lungs. A lower *n*-6 (linoleic acid) intake and higher *n*-3 (EPA + DHA) intake were associated with reduced odds of chronic cough and wheeze^(117)^.

A prospective study (421 309 individuals followed up for 16 years) showed that long-chain *n*-3 PUFA intake was significantly associated with 9 % lower total mortality, highlighting a 20 % reduction in respiratory disease mortality^(119)^. *n*-3 Fatty acids have been suggested as an adjuvant strategy in acute respiratory distress syndrome caused by COVID-19 uncontrolled inflammation^(19)^. However, a Cochrane review concluded that the benefits of EPA and DHA administration in patients with acute respiratory distress syndrome and antioxidants are uncertain due to the low quality of the evidence^(120)^. In this sense, *n*-3 fatty acids have been strongly associated with prevention tools in respiratory diseases and should be prioritised in regular dietary habits.

The primary sources of *n*-3 PUFA are fish (cod, halibut, mackerel, sardines and salmon), fish oils, flaxseed and chia, which are increasingly consumed by the population due to health benefits^(121,122)^.

### Phenolic compounds

The importance of phytochemicals against viral infections has been assessed. A recent randomised control trial demonstrated the effectiveness of the consumption of catechin-containing beverages for preventing acute upper respiratory tract infections^(123)^. Additionally, polyphenolic compound use has been suggested to manage influenza infection and its complications^(124)^.

Although most research has focused on isolated compounds, the synergistic effect of these compounds in food matrices can optimise the bioactivity of these compounds. There is a lack of studies concerning the safety profile of plant extracts and their isolated compounds^(125)^. Therefore, natural foods, mainly fruits and vegetables, are a good and safe option for improving the daily intake of phytochemicals.

Ferulic acid (4-hydroxy-3-methoxy cinnamic acid), according to the review by McCarty and DiNicolantonio^(114)^, acts in the amplification of TLR7 and mitochondrial antiviral signalling protein functions, which recognise viral nucleic acids and quickly trigger different signalling cascades. This action contributes to the production of interferons in antiviral defence. Thus, this beneficial health effect can help prevent and control infections by RNA viruses, including influenza and coronavirus. Ferulic acid is widely found in fruits and cereals^(126)^.

Studies have already investigated the effects of polyphenols, especially epigallo-catechin 3 gallate, in asthma and other lung diseases, such as COPD and pulmonary pneumonia, which can suppress inflammation and inflammatory cell infiltration in the lungs of asthmatic animals. This effect can also contribute to mitigating the inflammation caused by obesity, leading to other inflammatory processes such as asthma^(64)^. Besides, flavonoids can inhibit the influenza virus and the signalling of like receptor-type receptors, preventing NF-κB translocation. As a result, it is interesting to insert sources of these phytochemicals into the diet to strengthen the immune system and help avoid COVID-19. The primary sources of flavonoids are red wine, oranges, red fruits and vegetables and polyphenols are green tea, broccoli and apples^(116)^.

### Fibres

A recent study assessed the relationship between low fibre intake and respiratory morbidities and inflammation among adults who participated in the 2007–2012 National Health and Nutrition Examination Survey. Data from more than 13 000 individuals (20–79 years) showed a correlation between lower fibre intake and increased odds of asthma, as well as wheeze, cough and phlegm production. In the study, C-reactive protein was 1·6 times higher in the low fibre group than in the high fibre group^(127)^.

The literature has reported that a diet high in saturated fats and low in fibres can contribute to the pathology of asthma^(128)^, and the Centers for Disease Control and Prevention has considered that people with moderate to severe asthma may be at higher risk to COVID-19^(129)^.

Glucans, glucose polymers, are soluble and form the yeast cell wall, fungi, seaweed and cereals, such as oats. They have numerous beneficial biological effects on health, such as improving immune activity against infections, inhibiting cancer growth and reducing stress and cholesterol levels^(130,131)^. Vetvicka & Vetvickova^(130)^ used a murine model with an influenza virus that was treated for 2 weeks with an oral diet containing a mixture of glucans and evaluated phagocytosis, and cytokine levels, antibody response and cytotoxicity. They found that supplementation improved phagocytic activity in mice, restoring it to almost normal levels. The results showed a significant reduction in total mortality by influenza.

### Probiotics

According to a systematic review by Jayawardena *et al.*^(85)^, probiotics also play an important role in health. Studies show that probiotics have been effective in improving immunity in general, stimulating the production of antibodies. Strains of *Lactobacillus*^(85)^ and strains of *Bifidobacterium*^(132)^ alone or in association with vitamin C^(133)^ are recommended to prevent viral infections such as influenza. These effects also depend on eating an adequate and balanced diet.

In this context, Morais *et al.*^(134)^ through a narrative review highlighted the use of *Lactobacillus gasseri* in association with a diet rich in vitamins, minerals and bioactive compounds as an additional nutritional therapy to promote the immune system against viral infections, as in the case of SARS-CoV-2 infection, especially in patients with hyperuricaemia. This critical action of probiotics, especially *L. gasseri*, occurs because the microorganisms act on purine metabolism, reducing the availability of purine nucleotides for viral RNA and DNA synthesis.

## The relation between dietary patterns, epigenetic modifications, inflammation and immune function

Kinetic evidence shows that epigenetic remodelling plays a central role in mediating the transcriptional programme for activation of immune cells and immune memory, allowing the diet to influence immune function^(135)^. It is also worth remembering that genetic encoding is related to the individual response to diets, and this specificity must differentiate recommendations and nutritional guidelines.

Inflammatory dysregulation is related to epigenetic modifications, and it could be controlled through genetic, dietary and pharmacologic approaches^(136)^. Evans *et al.*^(137)^ discussed that epigenetic modifications usually modulate gene expression independently of modifying the DNA sequence. These epigenetic modifications by catalytic enzymes are responsible for adding or removing DNA proteins and histones, including DNA/histone methylation or acetylation^(137)^. Thus, epigenome-wide association studies have correlated dietary patterns and increased inflammation.

However, several inflammatory signalling pathways are regulated in part by epigenetics and diet via lifestyle. Natural foods can modulate metabolites from the energetic metabolism, such as S-adenosylmethionine, co-enzyme acetyl A and adenine nicotinamide dinucleotide. These metabolites regulate the differential acetylation and methylation status, which can directly influence gene expression and, consequently, induce epigenetic alterations^(137)^.

**Fig. 1.**
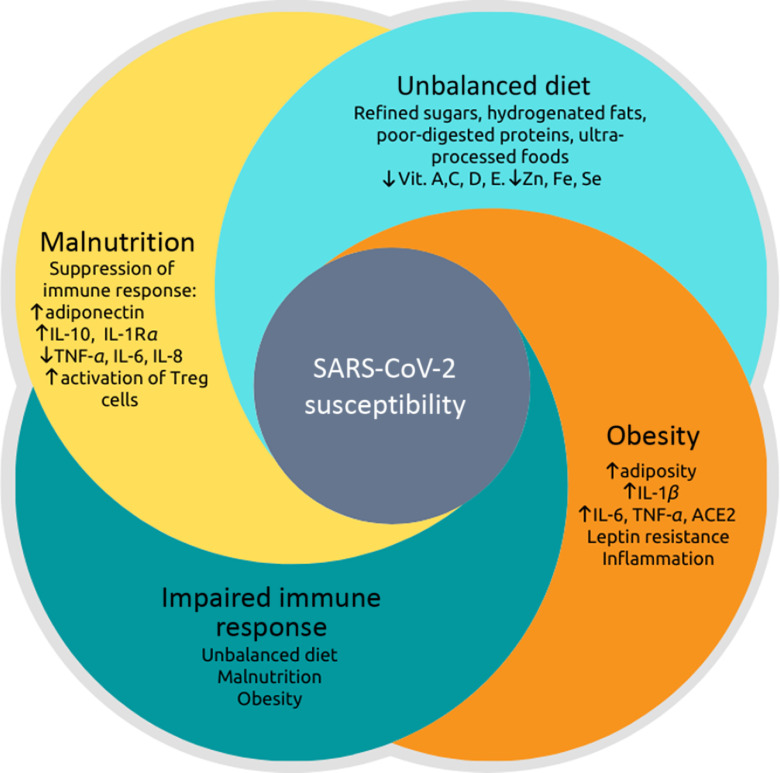
Relationship between malnutrition, dietary imbalance, obesity and impaired immune response, leading to greater susceptibility to severe acute respiratory syndrome coronavirus 2 (SARS-CoV-2) infection. Nutritional status is related to inflammation so that both malnutrition and obesity alter the innate and adaptive immune responses, increasing the risk of infections by various pathogens^(17–19,22,31–33)^. There is a synergic vicious cycle where infections induce a response that produces fever, increased catabolism, loss of appetite and altered intestinal absorption. These changes increase nutritional demands and, in addition to the loss of appetite, induce or aggravate malnutrition^(41,43)^. Otherwise, pro-inflammatory cytokines (TNF-*α*, IL-6 and IL-8) that act to fight pathogens are produced to a lesser extent in malnutrition, while anti-inflammatory cytokines, such as IL-10 increase^(47)^. The post-infection prognosis of obese individuals is worse^(53,55)^ because there are higher expressions and productions of angiotensinogen, angiotensin-converting enzyme 2 (ACE2), leptin and pro-inflammatory cytokines: TNF-*α*, IL-6 and IL-1*β*^(18,52,53)^. The dietary imbalance caused by the consumption of refined sugars, hydrogenated fats, poorly digested allergenic proteins, processed and ultra-processed foods increase the inflammatory index of the diet and has been associated with respiratory infection of the airways^(21,64,75–77)^. Besides, this dietary pattern provides a small content of vitamins (especially vitamins A, C, D and E) and minerals (iron, zinc and selenium), which can be deficient in situations of malnutrition and obesity^(16,69)^. These vitamins and minerals are also essential for immunocompetence against infections.

Therefore, dietary patterns can contribute to epigenetic modifications that can either promote or prevent metabolic diseases. However, nutritional interventions that modulate these factors through epigenetic pathways and their relationship with viral infections need to be explored. In this context, Fig. 1 represents the relationship between malnutrition and obesity on immune response impairment, considering the inflammatory potential influenced by food choices and their impact on susceptibility to SARS-CoV-2 infection. This figure summarises the main points highlighted in this review, showing that the pro-inflammatory dietary pattern provides a low content of essential vitamins and minerals, contributing to SARS-CoV-2 infection^(16,69)^.

## Conclusion

A diversified diet with a broad profile of nutrients and not just a specific nutrient or food can prevent and even reduce the vulnerability of patients with acute and chronic diseases during COVID-19. Healthy eating habits, micronutrients, bioactive compounds and probiotics can be adjuvant agents to confront COVID-19. Given the relationship between inflammation and viral respiratory infections and the relation with nutritional status and food choices, this review presented perspectives for the prevention and improvement of the inflammatory and immune status in viral infections, emphasising COVID-19. This knowledge is relevant, especially considering the exacerbated response caused by the cytokine storm in severe cases.
